# Optimal pseudorandom sequence selection for online c-VEP based BCI control applications

**DOI:** 10.1371/journal.pone.0184785

**Published:** 2017-09-13

**Authors:** Jonas L. Isaksen, Ali Mohebbi, Sadasivan Puthusserypady

**Affiliations:** 1 Department of Electrical Engineering, Technical University of Denmark, Kongens Lyngby, Denmark; 2 Laboratory of Experimental Cardiology, Department of Biomedical Sciences, University of Copenhagen, Copenhagen, Denmark; University of Minnesota, UNITED STATES

## Abstract

**Background:**

In a c-VEP BCI setting, test subjects can have highly varying performances when different pseudorandom sequences are applied as stimulus, and ideally, multiple codes should be supported. On the other hand, repeating the experiment with many different pseudorandom sequences is a laborious process.

**Aims:**

This study aimed to suggest an efficient method for choosing the optimal stimulus sequence based on a fast test and simple measures to increase the performance and minimize the time consumption for research trials.

**Methods:**

A total of 21 healthy subjects were included in an online wheelchair control task and completed the same task using stimuli based on the m-code, the gold-code, and the Barker-code. Correct/incorrect identification and time consumption were obtained for each identification. Subject-specific templates were characterized and used in a forward-step first-order model to predict the chance of completion and accuracy score.

**Results:**

No specific pseudorandom sequence showed superior accuracy on the group basis. When isolating the individual performances with the highest accuracy, time consumption per identification was not significantly increased. The Accuracy Score aids in predicting what pseudorandom sequence will lead to the best performance using only the templates. The Accuracy Score was higher when the template resembled a delta function the most and when repeated templates were consistent. For completion prediction, only the shape of the template was a significant predictor.

**Conclusions:**

The simple and fast method presented in this study as the Accuracy Score, allows c-VEP based BCI systems to support multiple pseudorandom sequences without increase in trial length. This allows for more personalized BCI systems with better performance to be tested without increased costs.

## Introduction

Brain Computer Interface (BCI) can be defined as the interaction between the brain and outside world through an external device, with the support of a computer. It operates by first extracting the brain signals (with electrodes), followed by processing these signals, in order to control an external device. It does not involve the usual output pathways of peripheral nerves [1]. Expressing it differently, a BCI system can be perceived as a communication scheme for the user to convert his/her intention to an actual action. Research within BCI has opened numerous encouraging options for people with disabilities suffering from Amyotrophic Lateral Sclerosis, Multiple Sclerosis, Acute Inflammatory Demyelinating Polyradiculoneuropathy, Spinal Cord Injury and more [2, 3]. In other words, BCI systems have shown to have a huge impact on the quality of life of people with such disabilities [4].

BCI systems based on electroencephalogram (EEG) have proved to be very useful and convenient because of their non-invasive nature and easy usability [5]. Different BCI schemes and approaches have been developed based on different types of EEG signals such as Event Related Potentials, Steady-State Visual Evoked Potentials (SS-VEPs), Slow Cortical Potentials, and pseudorandom code modulated VEPs (c-VEPs) [6–10]. In comparison with other modalities, c-VEP has achieved remarkable results with respect to the Information Transfer Rate (ITR), accuracy, and for providing the use of many targets in applications used [6, 7, 11], and multiple newer studies focus on c-VEP BCIs [12–14]. Particularly, c-VEP based BCI systems have been shown to outperform SS-VEP for robotic device control [15]. Independently of modality, BCI systems can have a wide range of applications from low-level binary applications to high-level applications with many degrees of freedom such as robotic arm control or drone control [16, 17]. In the present study, a simple wheelchair setup with four directions available was used. More advanced device control use a constant movement term which enables much faster task completion [17], but such a term was left out of the present application to simplify the task.

c-VEPs can be perceived as repetitive potentials elicited in the occipital lobe of the brain, when the eyes are presented to a certain visual stimulus flickering repeatedly in a pseudorandom pattern [7]. The average of the elicited potential repetitions often make up the template for one specific target. Templates for other targets are obtained by time-shifting the first template, corresponding to the shift in the initial pseudorandom code applied. An identification and classification of a target can be made by comparing the online EEG signals with the predefined templates [6].

It is clear from a 2013 review that the validation of BCI systems is often performed with only few test subjects [18]. In the specific review, out of 13 papers, the maximum number of participants was 14 and six papers (46%) had less than five participants! The low number of subjects in many BCI studies might reflect a limited amount of resources available for some research groups in terms of time or money, or both. If researchers are already struggling with including enough participants, for one reason or another, it seems clear that the trial should not be repeated *n* times for *n* different pseudorandom sequences in an *n*-times multi-code c-VEP based BCI system. This would be an argument against using many different pseudorandom sequences in a multi-code c-VEP based BCI system, favoring single-code systems. Clearly, a simple measure to predict the one best sequence for any particular subject is warranted.

In this study, the aim was to investigate the performance of different pseudorandom sequences in a c-VEP BCI and to present an easily obtainable measure for selecting the optimal pseudorandom sequence for a given subject.

## Materials and methods

### Population and session

A total of 21 healthy subjects with normal or corrected-to-normal vision were included in the present study. Sixty-two percent of the volunteers (*n* = 13) were male and the mean age (±SD) was 24.3 (±5.5) years. All subjects provided verbal informed consent prior to their participation in the experiments, which were approved by the Regional Committee on Health Research Ethics for the Capital Region of Denmark (reference H-3-2013-004) and carried out in accordance with the corresponding guidelines and relevant regulations on the use of human subjects for health-related scientific research.

Each session took about an hour to complete. First, the scalp skin was prepared using scrub gel (Matas Fodscrub, art. nr. 640457, Matas A/S, Denmark) and alcohol pads (Cutisoft Wipes, BSN Medical GmbH, Germany) after which the electrodes were placed on the scalp using conductive gel (Ten20 Conductive Paste, art. nr. 10-20-8, Weaver and Company, CO, USA). Next, the system was trained to recognize the response for a given stimulus, and thresholds were established. The subject was then trained in operating the system until the subject felt comfortable operating the system. Finally, the subject attempted to complete the task (testing). The training and testing were both repeated for each pseudorandom code.

### Experimental setup

**Fig 1:**
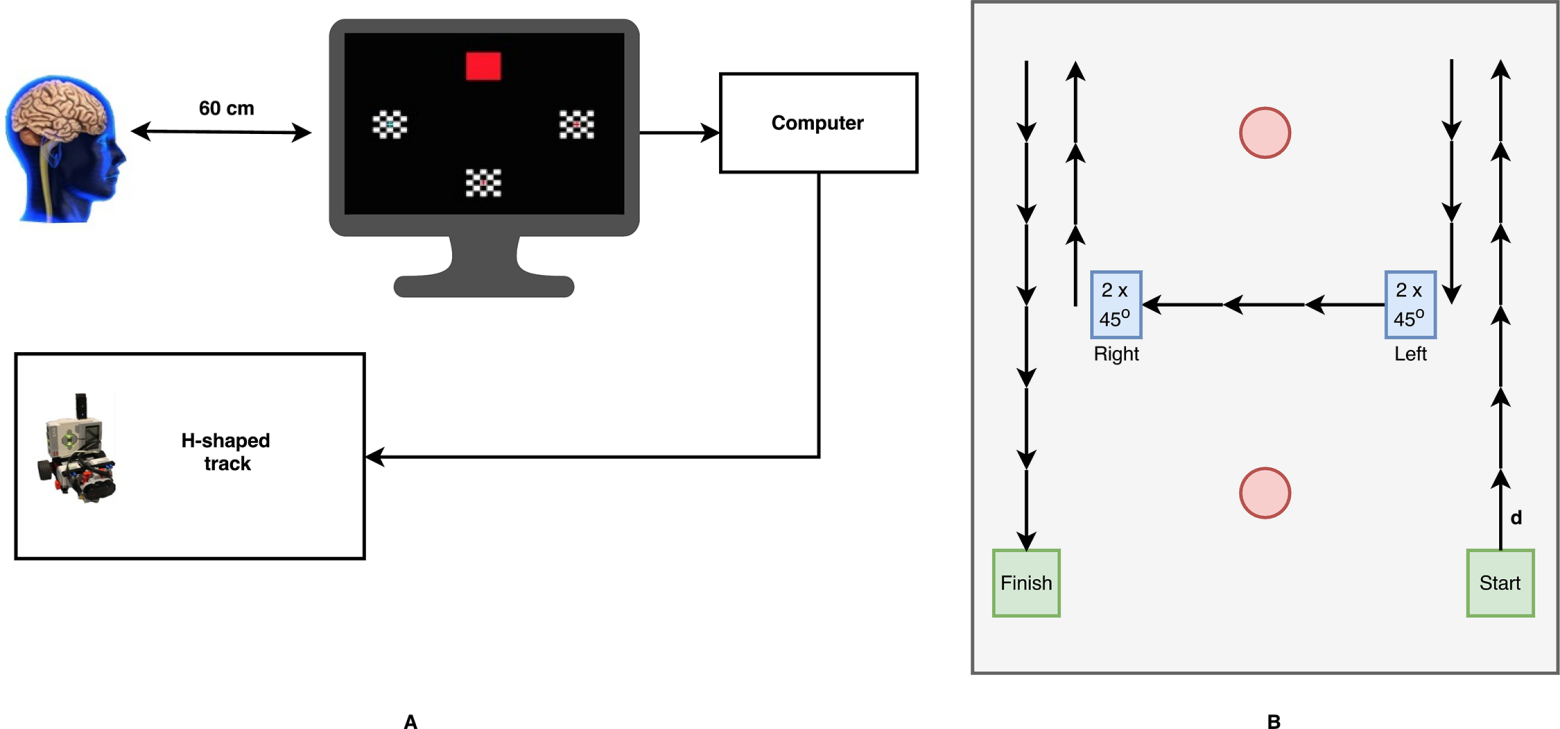
Experimental setup. *a)* Schematic overview of the c-VEP BCI system. *b)* The intended route consists of 25 steps: six steps forward, three steps backward, two turns left, three steps forward, two turns right, three steps forward, and six steps backward.

The subjects were seated at a distance of about 60 cm from the eyes to the monitor with full overview of the robot and its track, see Fig 1*A*. The monitor had a frame rate of *f*_*r*_ = 60 Hz. The track was H-shaped as depicted in Fig 1*B*. The track has soft boundaries such that any deviation from the track must be countered by a move in the opposite direction. No physical collision with surroundings occurred. The wheelchair model could move a fixed distance, *d*, either forward or backwards, or turn by 45 degrees either left or right. The two parallel sides of the H (track) were six *d* long, and the short side was three *d*, as shown in Fig 1. The intended route consisted of 25 steps: six *d* forward, three *d* backward, two 45-degree turns left, three *d* forward, two 45-degree turns right, three *d* forward, and six *d* backward, and the volunteer received real-time instructions to minimize the aspect of decision-making. Commands were transferred to the wheelchair model using WiFi.

### Individual measures

For each test subject and each code, primary (*T*_*p*_) and secondary (*T*_*s*_) thresholds were calculated for use in the classification algorithm. The primary threshold (*T*_*p*_) was defined as (1):

$$T_{p}=\frac{\alpha }{n_{t}n_{r}}\sum_{i=1}^{n_{t}}\sum_{j=1}^{n_{r}}R\left(y_{ij},{\bar{y}}_{i}\right)$$ (1)

whereby *R* denotes the Pearson’s product-moment correlation coefficient given in (2), *y*_*ij*_ is the *j*^th^ average response to the *i*^th^ target, ${\bar{y}}_{i}$ denotes the average response to the *i*^th^ target, *n*_*t*_ is the number of targets, *n*_*r*_ is the number of repetitions recorded for each target, and *α* = 0.8 is a thresholding constant based on the work in [19], included to accept some variation in the responses.

$$R(X,Y)=\frac{\sum_{n=1}^{N}\tilde{X}(n)\tilde{Y}(n)}{\sqrt{\sum_{n=1}^{N}{\tilde{X}}^{2}(n)\sum_{n=1}^{N}{\tilde{Y}}^{2}(n)}}$$ (2)

whereby $\tilde{X}(n)=X(n)-\frac{1}{N}\sum_{n=1}^{N}X(n)$ and *N* is the number of samples in the vectors *X* and *Y*. This normalized result thus gives a value between -1 [*R*(*X*,*-X*)] and 1[*R*(*X*,*X*)].

The secondary threshold (*T*_*s*_) was defined as (3):

$$T_{s}={\beta \;T}_{p}$$ (3)

whereby *β* = 0.625 is a scaling factor to lessen the thresholding criteria. The value was found empirically in [19] and used in this study as well.

Based on the Pearson’s correlation coefficient and a single target, we defined a measure, which we in this article shall denote the template consistency (*TC*), as defined by (4):

$$TC=\frac{1}{n_{R}}\sum_{i=1}^{n_{R}}R(x_{i},\bar{x})$$ (4)

whereby *R* is given by (2), *x*_*i*_ is the response to one repetition of code, $\bar{x}$ is the average of all responses, and *n*_*R*_ is the number of responses recorded. *TC* can attain values between -1 and 1. We used a number of responses corresponding to 12 seconds.

Master templates were acquired separately for each pseudorandom sequence and subject. A template for each target was acquired by circularly shifting the master template. For each new target, the template was shifted 30 samples corresponding to 3 bits in the pseudorandom code.

Based on the autocorrelation function (ACF) for the template, *T*(*n*), we defined the measure template periodicity (*TP*) according to (5) as the maximum value of the ACF at shift points, that is; points corresponding to other targets:

$$TP=\max_{i=1..n_{t}-1}ACF_{T}(i\frac{f_{s}}{f_{r}}n_{s})$$ (5)

Whereby *f*_*s*_ is the sampling frequency, *f*_*r*_ is the frame rate with which the sequences were cycled through, *n*_*s*_ is the number of samples that the sequence was shifted per new target and *ACF*_*T*_ is the circular ACF of the template, *T*.

### BCI system setup

A single lead system was applied using three Ag/AgCl electrodes. Electrode placements were defined using the international 10–20 system, and the active channel was placed on the Oz location, the reference was placed on Pz, and the ground was placed on Fpz. Impedances were kept below 5 kΩ. EEG was acquired at a rate of *f*_*s*_ = 600 Hz and band pass filtered at 5–30 Hz using an eighth order Butterworth filter. An additional notch filter was applied against 50 Hz interference.

#### Training session

Throughout the session, an order for the various codes was used, which was assigned at random for each subject. Templates were generated using a number of repetitions corresponding to a stimulus duration of 12 seconds (48 for sequences of length 15, and 55 for the sequence of length 13). The *TC* was obtained simultaneously. The template for a new target was found by shifting the template for the previous target by 30 samples corresponding to *n*_*s*_ = 3 bits.

Thresholds were generated according to (1) and (3) using *n*_*r*_ = 4 repetitions for *n*_*t*_ = 4 targets and stimulus times of 2 seconds.

Finally, the subject was allowed to control the robot without a fixed track until they felt comfortable with the system. The sequence with the highest *TC* was used for this part.

#### Interface and test session

Four targets (forward, backward, left, and right) consisting of a black and white 3x3 square checkerboard pattern of size at least 25 cm^2^ and a centered arrow were placed on a monitor. EEG was evaluated every two seconds (stimulus time) as correlation coefficients using (2) with the templates for each target. Upon successful classification, 1.5 seconds of visual feedback was given before stimulation resumed. The wheelchair model gave audio feedback in the form of a beep. The subjects had full visibility over the wheelchair model and its track and had audio instructions available. The instructor kept track of the correct and incorrect classifications, and the volunteer was instructed to notify the instructor in the rare event that he or she was looking at the wrong target.

Subjects were asked to keep going until completion or until one of two stopping criteria was reached. The two stopping criteria were 10 wrong classifications (18 cases) and no classification for 60 seconds (3 cases). The order of pseudorandom sequences in the training session was also applied in the test session.

#### Classification

**Fig 2:**
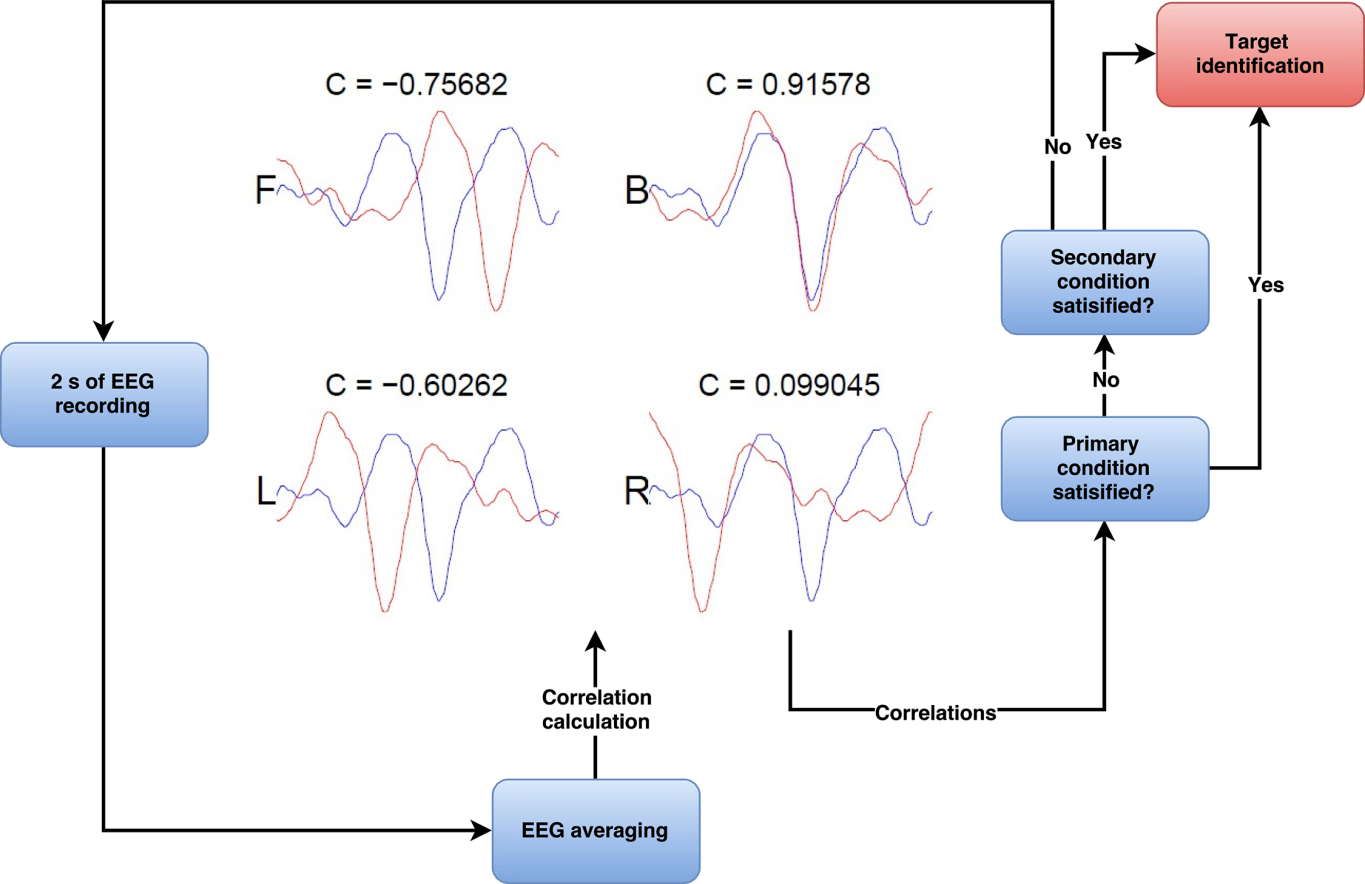
Classification algorithm. The two-step algorithm considers one or two blocks of two-second EEG at a time. The correlation coefficients from newly acquired EEG with known templates were used as features in the classification. Upon classification, the procedure repeats and two new seconds of EEG were recorded.

Classification of a target is based on the correlation coefficients obtained by comparing the online EEG recording with the predefined templates of each target. In Fig 2 the classification algorithm is illustrated. It starts with two seconds of continuous EEG recording, while running stimuli. Here, c-VEPs were produced followed by averaging the c-VEPs into one signal. This signal was correlated with the templates of each target, calculating and creating four feature values. These values were then tested against the primary and secondary conditions, in order to classify the right target. It took only one of the two conditions to determine which direction the subject was looking at. The primary condition was met if any feature value exceeded the primary threshold (1), and the target having the highest feature value was selected and a command was sent to the wheelchair model. If this condition was not met, the secondary condition was considered. At this point, the last two sets of feature values were summed into four new values. In the same manner, if one of these values were higher than the secondary threshold (3), that corresponding target was chosen as target. If none of the two conditions were met, additional EEG recording was performed for two seconds and classification was restarted.

#### Pseudorandom sequences

Three pseudorandom sequences (Table 1) were included in this study, namely the m-code, gold-code and the Barker-code. The former two were included with a length 15 and the latter with a length 13. For a 4-target system, these are reasonable lengths of code because they allow for a shift length of *n*_*s*_ = 3 bits. The m-sequence is the most widely-used pseudorandom sequence for BCI applications [6, 11–13] so it was natural to include this sequence in the present study. The reason for the widespread use of the m-sequence must be ascribed to the low values for its ACF [20, 21]. A gold-code is based on m-sequences and additionally contains good cross-correlation properties [21]. The gold-code has also previously been used in BCI research and its relation to the m-sequence warrants its inclusion [19, 22]. The Barker-code, although not often employed for BCI, is a promising pseudorandom sequence for its great ACF properties [23]. For that reason, we have included the Barker-code in the present study over other possibilities such as the Kasami (also related to the m-sequence) or Huffman sequences [21, 24, 25]. We limited the number of included sequences to three, to avoid too long sessions. Long sessions logically could reduce the performance markedly due to tiredness, which is an issue even though the order of the sequence used differs among subjects.

**Table 1:** Pseudorandom sequence details.

m-code	1	0	1	0	1	1	0	0	1	0	0	0	1	1	1
Gold-code	0	1	1	0	0	0	0	0	1	1	0	1	1	1	1
Barker-code	1	1	1	1	1	0	0	1	1	0	1	0	1		

**Fig 3:**
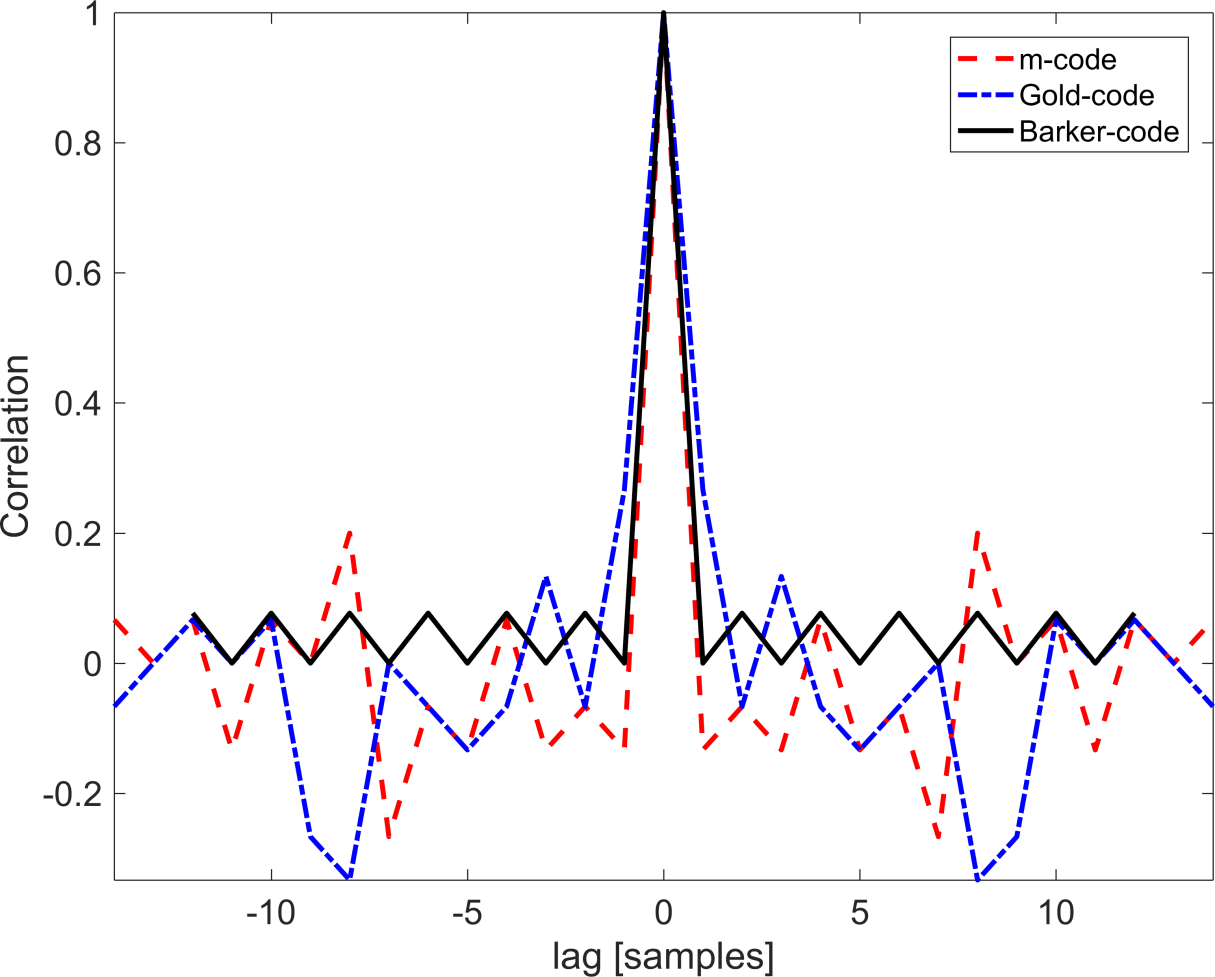
Autocorrelation functions. The ACFs of the m-code (red, dashed), gold-code (blue, dash-dot), and Barker-code (black, solid) all resemble delta functions.

The circular ACFs of each of the three sequences selected are depicted on Fig 3. The sequences have been selected based on the property that their ACFs resemble closely a delta function in the assumption that the response attains the same property to a high degree. The degree to which this assumption is met is quantified by the *TP* (5). c-VEP based BCI systems rely on being able to track the phase of the signal, which is straight-forward if the ACF of a signal resembles a delta function.

### Statistics

The accuracy of a given trial was defined by the ratio of correct classifications to total classifications. Further, each trial was marked as either completed or aborted; the completion rate is the ratio of completed trials to total trials. The commonly used ITR was also obtained for each run (equation given in [26]). ANOVA was used to identify whether one code was more accurate than the others, if one code had a higher ITR, and to identify whether one code had a higher completion rate than the others.

For each subject, the average accuracy and maximum accuracy were isolated. A two-sided t-test was used to assess whether the maximum accuracy was significantly larger than the average accuracy.

The trials for each subject were sorted by accuracy, and the average time per identification (TPI) was computed for each class. An ANOVA was used to test for differences in means.

The measures *TP* and *TC* were investigated as predictors for completion and accuracy in a first-order, forward step model. For the continuous variable (accuracy), a least squares fit was applied. For the categorical variable (completion), an ordinal logistic fit was applied.

A p-value less than 0.05 was in general considered significant, however, only a p-value less than 0.025 was considered significant when comparing three groups.

## Results

**Fig 4:**
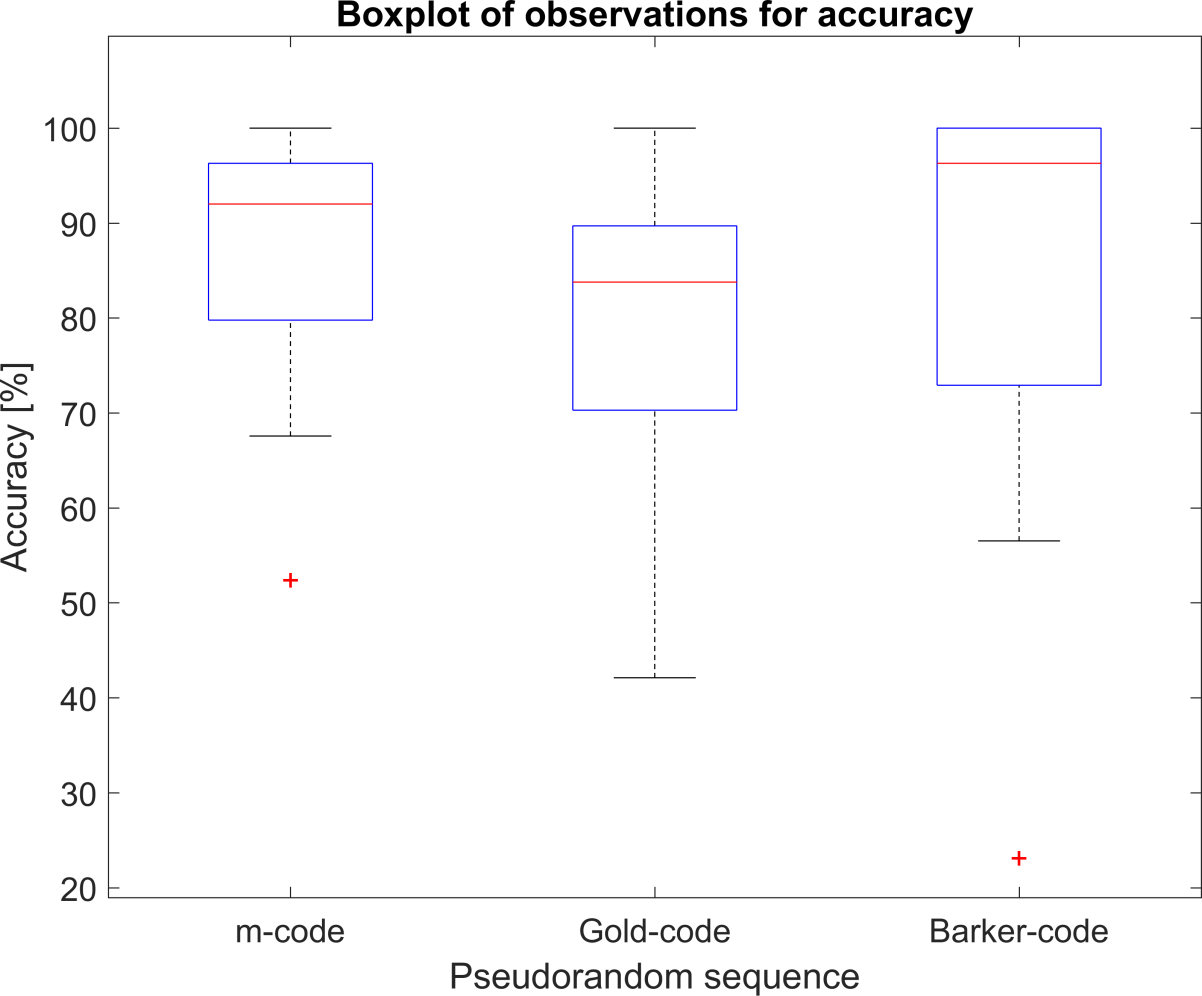
Boxplot of accuracy. The three boxes overlap to a high degree.

The accuracy, ITR, and completion rate for all trials with the three pseudorandom sequences are given in Table 2. The results show that no code is better than the others in terms of either accuracy, ITR, or completion rate (p>0.05). The boxplots of the accuracies for each code (Fig 4) show major overlap. On a group basis, no one code outperforms the others.

**Table 2:** Experimental results.

	m-code	Gold-code	Barker-code	p
**Accuracy** [%] (mean (SD))	87.2 (12.9)	78.4 (15.9)	85.7 (21.1)	NS(0.21)
**ITR** [bit/s] (mean (SD))	0.64 (0.3)	0.51 (0.3)	0.71 (0.35)	NS(0.1)
**Completion rate** (% (n))	71 (15)	62 (13)	67 (14)	NS(0.82)

NA: Not applicable. NS: Not significant. ITR: Information Transfer Rate.

Despite no significant difference on the group basis, it is possible that for each individual there is a gain to achieve by selecting the individually optimal pseudorandom sequence. To test this hypothesis, for each subject, we selected the top accuracy and the average accuracy of the three trials. The results are presented in Table 3. The top accuracy is significantly better than the average accuracy, and a large gain in accuracy of about 8.5 percentage points can be obtained by selecting the optimal code compared to the average performance.

**Table 3:** Benefit from choosing optimal sequence over average sequence.

	Top accuracy	Average accuracy	p
**Accuracy** [%] (mean (SD))	95.7 (6.8)	87.2 (9.9)	<0.001

To test for accuracy-TPI trade-off effects, for each subject, the trials were sorted by accuracy and the average TPI was computed. In Table 4, “Most accurate” represents the average TPI for the runs trials with the highest accuracy for each subject. No significant difference in TPI was found suggesting that no time is lost when optimizing for accuracy.

**Table 4:** Average TPI for trials sorted by accuracy.

	Most accurate	Median accurate	Least accurate	p
**TPI** [s] (mean (SD))	5.8 (5.2)	4.9 (2.8)	5.0 (3.1)	NS(0.67)

NS: Not significant. TPI: Time per identification.

Using the concepts of *TP* and *TC*, models were fitted to predict the accuracy of a trial and whether the trial would be completed. For completion prediction, only *TP* remained in the model (p<0.05, R^2^ = 0.10) suggesting that lower values of *TP* predict completion. For accuracy prediction, the final model (R^2^ = 0.16) consisted of both the *TP*, *TC*, and the interaction term (Table 5).

**Table 5:** Accuracy prediction.

	Estimate	t ratio	p
**Intercept**	80.3	8.78	<0.001
***TC***	14.5	0.80	NS(0.43)
***TP***	-29.3	-2.02	0.048
**(*TP*-0.481) ∙ (*TC*-0.123)**	-237.4	-2.01	0.049

NA: Not applicable. NS: Not significant. *TC*: Template consistency (4). *TP*: Template periodicity (5).

Using the estimates from the accuracy prediction model, the predicted accuracy is given by:

Accuracy=80.3+14.5∙TC−29.3∙TP−237∙(TP−0.481)∙(TC−0.123) (6)

As the intended use of the model is sequence selection, the intercept can be dropped, since it has the same value for all sequences. The resulting Accuracy Score *(AS)* (7) is obtained after dropping the intercept, carrying out the multiplication, and simplifying (6):

AS=43.8∙TC+85.0∙TP−237∙TC∙TP (7)

For a given subject, the pseudorandom sequence with the highest AS is predicted to yield the best performance.

## Discussion

An increase in accuracy can be found by selecting the optimal code without compromising the speed of the BCI system, and an indication as to which code is better for a given subject is given in this paper based on simple and easily obtainable measures.

### No one better pseudorandom sequence

We found that none of the three investigated pseudorandom sequences performed better than the others in terms of accuracy, ITR, and completion on a group basis. Sato et al. compared different pseudorandom sequences, but their off-line study focused on averaging of the responses and further used sequences of lengths 32 and 63 [27]. Thielen et al. also consider whether one pseudorandom code is better than other codes, but their focus is not on comparing pseudorandom codes [22].

Many metrics can be used for comparison of BCI systems and user performances besides the accuracy, ITR and completion rate which were used in this paper. Bianchi et al. [28] proposed the efficiency metric which better takes into account error-correction in BCI applications [29]. Dal Seno et al. [30] proposed the Utility metric, which takes a user-centered approach to evaluation and correctly estimates the bitrate as 0 if the accuracy is below 50% (due to correction of errors). Many other adaptations of the simple accuracy metric exists, for instance the projected accuracy [31].

### The optimal code is better than the average

Selecting the optimal pseudorandom sequence for each subject proved to be significantly better than the average sequence performance. This shows, that although on a group basis no sequence was better than the others, on the individual level it is highly beneficial to use different sequences for different individuals. This finding suggests that multi-code c-VEP BCI systems will outperform single-code c-VEP BCI systems, since each subject can use the code that performs the best in the individual case. It is well-known that a trade-off exists between accuracy and TPI [32]. However, we found no statistically significant decrease in TPI when selecting the code with the highest accuracy for each subject. Performance-wise, the multi-code system is superior to the single-code system. However, as described earlier, this requires that the optimal code can be selected without running full trials.

### How to select the optimal code

The optimal code can be selected using the simple measures *TC* (4) and *TP* (5) through the accuracy score, *AS* (7). For each subject, only the template along with the template consistency (*TC*) must be obtained. In our setup, this amounted to 12 seconds of stimulation per code, which means that the code selection can be done within minutes for systems that support many pseudorandom sequences. The template periodicity measure, *TP*, can be readily computed from the template directly. The investigator can then select for each subject the pseudorandom sequence that has the highest *AS*. In this way, the system gains the advantages of speed from the single-code system and the performance from the multi-channel system because the full trial must not be run with all supported pseudorandom sequences, but only with the best one for the particular user.

### Limitations

The present study deals with pseudorandom sequences of relative short length and with few targets. While the inclusion of 21 subjects is a relatively high number for BCI studies, a new, prospective study should be performed, that evaluates the suggested approach of using *AS* (7) to select the optimal pseudorandom sequence. More importantly, only healthy subjects were included in the present study, and the system as well as the selection method should be tested in patients.

The wheelchair application is a relatively high-level application for a trial that is the first of its kind. Although the study was highly controlled in its setup, an online study with a simpler setup is called for to validate the concept.

## Conclusions and outlook

The present study showed the superiority of multi-code c-VEP BCI systems over their single-code counterparts and presented a simple and efficient method for selecting the optimal pseudorandom sequence in a fast way.

Implementing more than one pseudorandom sequence into a c-VEP BCI system is no great effort, but brings a huge advantage. It is our firm recommendation that future c-VEP BCI systems be built to support multiple pseudorandom sequences to account for biological differences in subjects.

## Supporting information

S1 Table Full dataset. (XLSX)

## References

[1] Wolpaw JR, Birbaumer N, McFarland DJ, Pfurtscheller G, Vaughan TM. Brain-computer interfaces for communication and control. Clin Neurophysiol. 2002;113(6):767–91. . 12048038 10.1016/s1388-2457(02)00057-3

[2] Millán JdR, Rupp R, Mueller-Putz G, Murray-Smith R, Giugliemma C, Tangermann M, et al Combining Brain–Computer Interfaces and Assistive Technologies: State-of-the-Art and Challenges. Frontiers in Neuroscience. 2010;4(161). doi: 10.3389/fnins.2010.00161 20877434 10.3389/fnins.2010.00161 PMC2944670

[3] Rao RPN. Brain-Computer Interfacing: An introduction: Cambridge University Press; 2013.

[4] Wolpaw J, Wolpaw EW. Brain-computer interfaces: principles and practice: OUP USA; 2012.

[5] Shih JJ, Krusienski DJ, Wolpaw JR. Brain-computer interfaces in medicine. Mayo Clin Proc. 2012;87(3):268–79. doi: 10.1016/j.mayocp.2011.12.008 ; PubMed Central PMCID: PMCPMC3497935. 22325364 10.1016/j.mayocp.2011.12.008 PMC3497935

[6] Bin G, Gao X, Wang Y, Hong B, Gao S. VEP-based brain-computer interfaces: time, frequency, and code modulations [Research Frontier]. IEEE Computational Intelligence Magazine. 2009;4(4):22–6.

[7] Nicolas-Alonso LF, Gomez-Gil J. Brain Computer Interfaces, a Review. Sensors. 2012;12(2):1211–79. doi: 10.3390/s120201211 22438708 10.3390/s120201211 PMC3304110

[8] Zhu D, Bieger J, Garcia Molina G, Aarts RM. A survey of stimulation methods used in SSVEP-based BCIs. Comput Intell Neurosci. 2010:702357 doi: 10.1155/2010/702357 ; PubMed Central PMCID: PMCPMC2833411. 20224799 10.1155/2010/702357 PMC2833411

[9] Berger TW, Chapin JK, Gerhardt GA, McFarland DJ, Principe JC, Soussou WV, et al Brain-Computer Interfaces: An international assessment of research and development trends: Springer Science & Business Media; 2008.

[10] Hassanien AE, Az Ar AATA. Brain-Computer Interfaces: Current trends and applications: Springer; 2015.

[11] Bin G, Gao X, Wang Y, Li Y, Hong B, Gao S. A high-speed BCI based on code modulation VEP. J Neural Eng. 2011;8(2):025015 doi: 10.1088/1741-2560/8/2/025015 . 21436527 10.1088/1741-2560/8/2/025015

[12] Spuler M, Rosenstiel W, Bogdan M. Online adaptation of a c-VEP Brain-computer Interface(BCI) based on error-related potentials and unsupervised learning. PLoS One. 2012;7(12):e51077 doi: 10.1371/journal.pone.0051077 ; PubMed Central PMCID: PMCPMC3517594. 23236433 10.1371/journal.pone.0051077 PMC3517594

[13] Wei Q, Feng S, Lu Z. Stimulus Specificity of Brain-Computer Interfaces Based on Code Modulation Visual Evoked Potentials. PLoS One. 2016;11(5):e0156416 doi: 10.1371/journal.pone.0156416 ; PubMed Central PMCID: PMCPMC4886965. 27243454 10.1371/journal.pone.0156416 PMC4886965

[14] Isaksen J, Mohebbi A, Puthusserypady S. A comparative study of pseudorandom sequences used in a c-VEP based BCI for online wheelchair control. Engineering in Medicine and Biology Society (EMBC), 2016 IEEE 38th Annual International Conference of the. 2016:1512–5. 10.1109/EMBC.2016.7590997 28324945

[15] Kapeller C, Hintermuller C, Abu-Alqumsan M, Pruckl R, Peer A, Guger C. A BCI using VEP for continuous control of a mobile robot. Conf Proc IEEE Eng Med Biol Soc. 2013;2013:5254–7. doi: 10.1109/EMBC.2013.6610734 . 24110921 10.1109/EMBC.2013.6610734

[16] Meng J, Zhang S, Bekyo A, Olsoe J, Baxter B, He B. Noninvasive Electroencephalogram Based Control of a Robotic Arm for Reach and Grasp Tasks. Sci Rep. 2016;6:38565 Epub 2016/12/15. doi: 10.1038/srep38565 ; PubMed Central PMCID: PMCPMC5155290. 27966546 10.1038/srep38565 PMC5155290

[17] LaFleur K, Cassady K, Doud A, Shades K, Rogin E, He B. Quadcopter control in three-dimensional space using a noninvasive motor imagery-based brain-computer interface. J Neural Eng. 2013;10(4):046003 Epub 2013/06/06. doi: 10.1088/1741-2560/10/4/046003 ; PubMed Central PMCID: PMCPMC3839680. 23735712 10.1088/1741-2560/10/4/046003 PMC3839680

[18] Amiri S, Fazel-Rezai R, Asadpour V. A review of hybrid brain-computer interface systems. Advances in Human-Computer Interaction. 2013;2013:1.

[19] Mohebbi A, Engelsholm SK, Puthusserypady S, Kjaer TW, Thomsen CE, Sorensen HB. A brain computer interface for robust wheelchair control application based on pseudorandom code modulated Visual Evoked Potential. Conf Proc IEEE Eng Med Biol Soc. 2015;2015:602–5. doi: 10.1109/EMBC.2015.7318434 . 26736334 10.1109/EMBC.2015.7318434

[20] Golomb SW. Shift register sequences. Rev. ed ed. Laguna Hills, Calif.: Aegean Park Pr.; 1982 X, 247 S. p.

[21] Sarwate DV, Pursley MB. Crosscorrelation Properties of Pseudorandom and Related Sequences. Proceedings of the IEEE. 1980;68(5):593–619.

[22] Thielen J, van den Broek P, Farquhar J, Desain P. Broad-Band Visually Evoked Potentials: Re(con)volution in Brain-Computer Interfacing. PLoS One. 2015;10(7):e0133797 doi: 10.1371/journal.pone.0133797 ; PubMed Central PMCID: PMCPMC4514763. 26208328 10.1371/journal.pone.0133797 PMC4514763

[23] Golomb SW, Scholtz RA. Generalized Barker Sequences. IEEE Transactions on Information Theory. 1965;IT-11(4):533–7.

[24] Ackroyd MH. Synthesis of efficient Huffman sequences. IEEE Transactions on Aerospace and Electronic Systems. 1972;AES-8:2–6.

[25] Kasami T. Weight distribution formula for some class of cyclic codes ILLINOIS UNIV AT URBANA COORDINATED SCIENCE LAB, 1966.

[26] Wolpaw JR, Ramoser H, McFarland DJ, Pfurtscheller G. EEG-based communication: improved accuracy by response verification. IEEE Trans Rehabil Eng. 1998;6(3):326–33. . 9749910 10.1109/86.712231

[27] Sato J, Washizawa Y. Reliability-based automatic repeat request for short code modulation visual evoked potentials in brain computer interfaces. Conf Proc IEEE Eng Med Biol Soc. 2015;2015:562–5. doi: 10.1109/EMBC.2015.7318424 . 26736324 10.1109/EMBC.2015.7318424

[28] Bianchi L, Quitadamo LR, Garreffa G, Cardarilli GC, Marciani MG. Performances evaluation and optimization of brain computer interface systems in a copy spelling task. IEEE Trans Neural Syst Rehabil Eng. 2007;15(2):207–16. doi: 10.1109/TNSRE.2007.897024 . 17601190 10.1109/TNSRE.2007.897024

[29] Quitadamo LR, Abbafati M, Cardarilli GC, Mattia D, Cincotti F, Babiloni F, et al Evaluation of the performances of different P300 based brain-computer interfaces by means of the efficiency metric. J Neurosci Methods. 2012;203(2):361–8. doi: 10.1016/j.jneumeth.2011.10.010 . 22027493 10.1016/j.jneumeth.2011.10.010

[30] Dal Seno B, Matteucci M, Luca M. The Utility Metric: A Novel Method to Assess the Overall Performance of Discrete Brain-Computer Interfaces. IEEE Transactions on Neural Systems and Rehabilitation Engineering. 2010;18(1):20–8. doi: 10.1109/TNSRE.2009.2032642 20064766 10.1109/TNSRE.2009.2032642

[31] Colwell K, Throckmorton C, Collins L, Morton K Jr. Projected accuracy metric for the P300 Speller. IEEE Trans Neural Syst Rehabil Eng. 2014;22(5):921–5. doi: 10.1109/TNSRE.2014.2324892 . 25203496 10.1109/TNSRE.2014.2324892

[32] Standage D, Blohm G, Dorris MC. On the neural implementation of the speed-accuracy trade-off. Front Neurosci. 2014;8:236 Epub 2014/08/29. doi: 10.3389/fnins.2014.00236 ; PubMed Central PMCID: PMCPMC4131279. 25165430 10.3389/fnins.2014.00236 PMC4131279

